# Household Structure and Suburbia Residence in U.S. Metropolitan Areas: Evidence from the American Housing Survey

**DOI:** 10.3390/socsci5040074

**Published:** 2016-11-17

**Authors:** Gowoon Jung, Tse-Chuan Yang

**Affiliations:** Department of Sociology, University at Albany, State University of New York, 351 Arts & Sciences Building, 1400 Washington Avenue, Albany, NY 12222, USA

**Keywords:** residential attainment, household structure, suburban residence, hierarchical modeling

## Abstract

Suburbs have demographically diversified in terms of race, yet little research has been done on household structures in suburbs. Using the 2011 American Housing Survey and 2009–2013 American Community Survey, this study investigates the distributions of household structures in suburbia and central cities, and the relationship between household structures and residential attainment. The findings of this research include: (1) The distribution of household structures differs between suburbia and central cities. Married-couple households are the most common household type in both central cities and suburbs, but they are more likely to reside in suburbia than in central cities; (2) Household structure is a determinant of residential attainment and the relationship varies by race/ethnicity groups. Among Hispanics and Asians, multigenerational household structure is indicative of central city residence, but this association does not hold for whites and blacks. For multigenerational households, the odds of living in suburbia decreases by almost 40 percent among Hispanics and by almost 50 percent for Asians.

## 1. Introduction

The question of what makes people move has been the focus of research on residential mobility. The existing literature suggests that residential mobility can be understood with factors relating to different aspects. At the individual or family level, racial and socioeconomic demographic status are important predictors [1,2], and this is especially crucial for explaining spatial assimilation of minorities [3]. Suburban residence suggests a decent neighborhood environment, access to a range of amenities, and presence of middle-class role models. Studies have found that suburbs are diverse, including manufacturing suburbs and poor suburbs [4,5], and suburban residents have become demographically heterogeneous as shown by the presence of moderate and low income residents and black, Hispanic, and Asian minorities [4,6]. However, they are still more homogeneous than in the central city [7], implying the preservation of affluent lifestyle. Given these underlying meanings of suburbian residence, moving to suburbia thus may have long been translated into assimilation to the mainstream culture and desirable social status, particularly for minorities [3]. This line of inquiry further explores the determinants of suburban residence and several factors have been identified. For example, those with higher family income, better educational attainment and who are married and of white race are more likely to reside in suburbia [3]. Beyond the individual and family level factors, contextual variables, such as the housing market, housing supply and racial discrimination in the housing market, were found to be associated with suburban residence [3,8–10]. Other neighborhood and metropolitan factors include white avoidance of black neighborhoods [11–13], neighborhood socioeconomic conditions [14,15], and metropolitan features such as population size, proportion of racial minorities, metropolitan housing supply and industrial structure, and local levels of segregation [2,16].

Despite the extensive effort to understand why people move to suburbia, little is known about whether household structure plays a role in determining suburban residence. Previous studies were mainly focused on testing the effects of household income, education, occupation, race/ethnicity, nativity-status, and language proficiency on suburban residence [3,17–19]. Household structure has been largely overlooked even though it is closely related to residential status. The variation of family size and composition differentiates the needs for housing service, amenities, and location choice. For instance, some studies showed that extended families of racial minorities and immigrants (e.g., Mexican immigrant families) live in small and overcrowded dwellings in central cities, due to their easy access to ethnic communities and employment in ethnic enclaves [20]. By contrast, demographic studies report that nuclear families with children live in suburban areas, enjoying a peaceful and safe space for their children [21]. Despite several studies connecting household composition and living space, there has been little systematic work to examine how household structure is associated with residential status.

Furthermore, the U.S. has witnessed the family and household transition. The transition refers to the fact that the number of traditional families (i.e., married-couples with children) has rapidly shrunk [22] and the number of extended families has increased [23–26].^1^ The family and household transition is a consequence of several sociodemographic changes in the past few decades, such as economic recession, low fertility, delayed marriage, prolonged life expectancy, and international migration [23,24]. Particularly, it is noted that the rise in the number of immigrants during the 1970s and 1980s may explain the increase in the percentage of extended families among racial minorities and foreign born-immigrants [23]. Although the family and household transition has drawn much attention with respect to the implications for children’s well-being and gender equity [27], its association with residential attainment remains underexplored. Examining the household structure and residential attainment is important since it guides policy makers and practitioners to approach housing policies in light of family needs and household changes.

This study aims to fill this gap by examining the intertwined relationships among household structures, race/ethnicity, nativity status, and residential attainment. Specifically, we ask two interrelated research questions: (1) whether multigenerational families of minorities are more likely to live in suburbia than in central cities and (2) whether household structures play different roles in residential attainment for different races/ethnicity groups. Below we first elaborate on suburbanization and why household structure should be considered in residential attainment research and then propose research questions and hypotheses, followed by the discussion of data and methods. We will then present the results and discuss our findings at the end.

## 2. Background

### 2.1. Suburban Residential Attainment

In the late urban-industrial era, suburban growth was propelled by transportation and technology development such as mass-produced automobiles and highways [28]. Other socio-demographic changes after World War II such as the rapid increase of marriage rate and fertility rate contributed to the suburban expansion. Housing policy has influenced suburban expansion trends throughout history, and one key policy was the National Housing Act of 1934, the G.I. Bill^2^, and the Fair Housing Act [9]. Furthermore, urban problems such as high crime rates, unemployment, racial tensions, and rising taxes have been referred to as factors contributing to suburbanization. Government and industry promotion also played a central role in idealizing the suburban life as a quest for the American Dream [29]. In the beginning, the dominant suburban population was composed of white, family-oriented middle-class people who commuted to white collar jobs in the central city [30,31]. Those people who could afford the higher costs of housing and commuting to the central city moved to suburbia [32,33]. As a result, the suburban population in the U.S. increased dramatically, so the suburban population in the 1960 was almost equal to the central city population. Since 1960, the population living in suburbia has outnumbered people living in central city. By the year 2000, suburbs had continued to grow, to the extent that the half of the entire U.S. population lived in the suburbs [34].

Since the advent of suburbia, urban sociologists have presumed that suburbia is the ultimate destination for residential movement regardless of race and ethnicity. Spatial assimilation theories put importance on place as one of the important markers to measure the assimilation of racial minorities and foreign-born population [35]. Suburban residential attainment indicates that people successfully transformed the material accomplishment to housing. These notions led to a large body of research that explored the determinants of residential mobility and attainment. The following factors have been found to affect suburban residential attainment: socioeconomic status, level of segregation, racial discrimination in housing market, and neighborhood features in metropolitans [1,2,9,14–16]. These studies have largely paid attention to individual factors.

Beyond these well-documented determinants, Rossi [36] suggested that ‘family and households’ should be considered as a crucial factor in understanding residential mobility. According to Rossi [36], a household is an important unit of mobility, and household-related factors moderate residential mobility. For example, “household’s dissatisfaction with dwelling and moves due to marriage, divorce and employments” are considered as determinative conditions for the residential shifts to central city or suburbia ([36], p. 24). Furthermore, household housing needs are conditional on family life cycle, particularly different life events such as births, deaths, marriages, and divorces. That is, the change in family size or the composition of family/household members may alter the need for housing location and/or services. In other words, this perspective uses life-cycle changes and life course approaches to understand why household structures play a role in residential attainment. Households move due to changing housing needs created by different family composition throughout the life cycle [37,38].

Applying the life-cycle changes, prior studies have focused on several characteristics related to household heads or household compositions, such as the age or marital status of the household head and the number of children. Scant research has focused on the importance of the household structure in determining the residential location. In this paper, we build on this family life course perspective and investigate whether and why household structures matter for suburban residence.

### 2.2. Household Structure and Location

Families and household living arrangements in the U.S. have shifted over time due to changes in local labor markets, migration patterns, and economic recessions [39]. Despite the overlapping meaning between families and households, households cover a broader living arrangement than families. The definition of family is restricted by the presence of blood or marital ties linking the householder to other members of the household, while a household is often defined as a group of individuals sharing the same housing unit. Combining these two concepts, the U.S. Census Bureau (2012) categorizes households into family households and nonfamily households. A family household has at least two members related by birth, marriage, or adoption, to the householder, whereas a nonfamily household is either a person living alone or a householder who shares the housing unit only with nonrelatives. Furthermore, family types have been diversified recently such as the emergence of step families and cohabitating couples. This trend suggests a retreat from the traditional definition used to define a family (e.g., marriage and adoption). The concept of households clearly is broader than family and it is a unit that has been used to study the geography of family and household living arrangement [39].

Extending from the discussion above, households can be classified into four groups: (1) one-person households; (2) married-couple households; (3) multigenerational households; and (4) married-couple households with others. According to the Census Bureau (2012), one-person households and married-couple households with others belong to non-family households, while married-couple households and multigenerational households make up family households. Firstly, one-person households refer to those living alone in a housing unit. Secondly, married-couple households refer to couples by marriage with or without children present^3^. Thirdly, multigenerational households refer to family households consisting of three or more generations. These include families with either a householder with a parent and a child, a householder with both a child and grandchild, a householder with both a grandchild and a parent, or a four-generation household [39]. Lastly, married-couple households with others refer to couples by marriage with or without children living together with nonfamily members. These types of households include family living with friends, roommates, or others.

We argue that household structure is associated with where a household resides. The variation of family size and composition differentiates the needs for housing service and location choice. Classified as non-family households, one-person households tend to live in central cities. Young adults are known to reside in central city locations due to the neighborhood liveliness and temporary housing consumption patterns. Neighborhood environments such as “proximity to restaurants, mixed-used land uses, and night life” attract young householders. Also, “temporary housing needs” make young one-person householders remain in central city ([37], pp. 4–5).

In contrast, married-couple households tend to live in suburbia. Historically, suburbia has been portrayed as an ideal place for married-couple households that have children. The favorable living conditions of suburbia, the provision of safety, a peaceful and ample space, and physical comfort, meet parents’ expectation of raising children [21]. Middle-class whites distanced themselves from the poor and black residents in central cities. Suburbia was described as a proper place of achieving the American Dream through mass media [21]. Households in early married years, for example just-married and young married households, tend to reside in temporary homes near employment and education centers, however the birth of children under school age encourages them to settle in suburbia. A study focusing on the phases of household formation asserts that households with school-aged children demand higher housing consumption and mobility compared to just-married or young married households [37]. The higher rate of housing consumption behavior therefore results in suburban residential choice.

Multigenerational households, perceived to have been replaced by married-couple households, have recently returned due to socio-demographic changes such as delayed marriage, the lack of employment and housing for young people, and growing numbers of immigrants [23,25,26,40–42]. Given these factors, while multigenerational households are less common among whites, we expect that white multigenerational households are more likely to be found in suburbia as young adults move into their parents’ homes in the suburbs in order to live together. In contrast, multigenerational households are more common among racial minorities and foreign-born immigrants [23]. Coupled with the fact the ethnic enclaves/communities and minority groups are concentrated in central cities, it is reasonable to envision that the multigenerational households are more prevalent in central cities than in suburbia.

Two additional factors may further complicate the relationship between multigenerational households and locations. One is race/ethnicity. Specifically, housing attainment in suburbia may shift down or be barred for minority households for economic or other reasons. Racial minorities opt to establish multigenerational households to share resources for the purpose of alleviating poverty and income inequality [23,43–46]. It is likely that suburbia attracts married-couple households of racial/ethnic minorities who aspire to assimilate to the culture of whites, nonetheless, multigenerational households of racial/ethnic minorities may still stay in central cities due to economic and cultural reasons.

The other factor is one’s foreign-born status. Foreign-born individuals tend to establish multigenerational households and more often view central cities as an ideal location rather than the suburbs [47,48]. For example, multigenerational households in central cities can translate to close-knit social connections, and immediate help from relatives and acquaintances, which helps foreign-born households to survive and thrive. Several studies have found that immigrants from Mexico and other Latin American countries prone to building horizontally multigenerational households for temporary housing needs [23] and foreign-born households in New York City are more likely to live in overcrowded housing units, which implies their residence in central cities [49].

## 3. Hypothesis

The discussion above leads to two research questions. Firstly, while the racial/ethnic diversity in suburbia has been increasing, it is unclear whether multigenerational families of minorities prefer suburbia to central cities. Secondly, given the family and household transition in the past few decades, it remains underexplored whether household structures play a different role in residential attainment for different race/ethnicity groups. We focus on race/ethnicity variation because household structure, especially increasing multigenerational households, is more closely related to race/ethnicity.^4^ To answer these questions, we propose the following hypotheses:

(H1) The distribution of household structures differs between suburbia and central cities. Specifically,
  (H1a) One-person households are more prevalent in central cities than suburbia.
  (H1b) Married-couple households are more prevalent in suburbia than central cities.
  (H1c) Married-couple households with others are more prevalent in suburbia than central cities.
(H2) Household structure is a determinant of residential attainment even after accounting for other household characteristics and the relationship between household structure and residential attainment varies by race/ethnic groups. Specifically,
  (H2a) Overall, married-couple households remain more likely to reside in suburbia than in central cities and one-person households are more like to be found in central cities.
  (H2b) Among whites, multigenerational households are more likely to live in suburbia than other household structures.
  (H2c) Among blacks, multigenerational households tend to stay in central cities, in contrast to other household structures.
  (H2d) For non-black minorities, multigenerational households prefer to live in central cities instead of suburbia.

## 4. Data and Method

### 4.1. Household Level Data and Measures

This study uses data from the 2011 American Housing Survey (AHS) to examine the suburban residential distribution of multigenerational households and other household types in suburbia. The public use file for the 2011 American Housing Survey (AHS) contains 186,448 cases overall; however, this study extracted the cases (*n* = 88,600) from the 29 metropolitan areas and used the metropolitan level weights (included in the 2011 AHS) in the multivariate analysis of this study [50]. The main reason for using the samples in the 29 metropolitan areas^5^ is to further consider metropolitan factors that may affect residential attainment in the analysis, such as segregation and socioeconomic conditions.

The strength of the AHS data lies in the information available on the residential status of living, whether people live in central cities or suburban areas within a metropolitan or nonmetropolitan area. Also, it collects various household socioeconomic and demographic data. The AHS is a self-reported survey and interviews one person (not necessarily the head) in a household to collect information.

The outcome variable of interest in this paper is residential status, whether households dwell in the central city or in suburbia. Residential status indicates the place where households are, and its subcategories include central cities or suburbs in metropolitan areas. By definition, a suburb is defined as an area located at the edge of city. It is usually a residential area and dependent upon the city for employment and other benefits. Given that suburbs are historically developed by white middle class moving out from the central business district, the definition of suburbs only applies to metropolitan areas. In the analysis, the outcome variable was *suburban residence*, a dichotomous variable where suburbia is coded 1 and central city is coded 0.

The key independent variable in this paper is *household structure*. We followed the definitions by the U.S. Census Bureau to categorize families^6^ into four types: one-person households, married-couple households, multigenerational households, and married-couple households with others. A one-person household refers to a person living alone in a housing unit [39,51]. Married-couple households^7^ refer to couples by marriage with or without children present [22,39]. Multigenerational households^8^ refer to family households consisting of three or more generations [39]. This definition can include multigenerational household members such as siblings, parents, grandchildren, cousins, and other distant kin. The common example of multigenerational households is the multigenerational family which contains grandparents, couples and their children, and other relatives. A married-couple households with others represent a family where members are related other than blood relationships, or a family living with an unrelated subfamily or individual. Examples are the married-couple households living with friends or with leasing roommates or family. Three dummy variables were created in the analysis using married-couple households as the reference group.

In addition to household structures, three other groups of independent variables were considered. Firstly, in the 2011 AHS, *race/ethnicity* has six categories: non-Hispanic whites (*n* = 58,257), non-Hispanic blacks (*n* = 12,209), Hispanics (*n* = 11,591), Asians (*n* = 6072), Native Americans (*n* = 338), and non-Hispanic others (*n* = 133). When race/ethnicity^9^ is included in an analysis, non-Hispanic whites are used as the reference group. We also note that due to the small numbers of respondents, the multivariate results of Native Americans and non-Hispanic others will not be presented but they are available upon request. Second, socioeconomic status includes three variables, namely *educational attainment, home ownership*, and *household income*. Educational attainment is based on the head of a household and divided into four groups: less than high school (reference group), high school graduate, some college or college graduate, and post-college degree. Home ownership is coded 1 for those who own the housing unit, otherwise 0. Household income (in thousands) is transformed with natural logarithm and then included in the analysis. The last group covers a range of demographic features. *Household head* is coded as 1 if the interviewee is the head, otherwise 0; those who are *married* are coded 1 and other marital statuses are all coded 0. Another dichotomous variable is *nativity status* in which the foreign-born are coded 1, otherwise 0. A respondent’s *age* and the *total number of children* in a household are both treated as continuous variables.

### 4.2. Metropolitan Level Data and Measures

As our samples came from the 29 metropolitan areas in the 2011 AHS, the differences across metropolitan areas, such as housing market and economic performance, may confound the relationships between household structures and suburban residence. To address this issue, we used a multilevel analysis approach to adjust for the clustering effects. We extracted the following metropolitan level variables from the 2009–2013 American Community Survey (centered on 2011): percent of owner-occupied housing units, percent of population who did not move in the past year, poverty, and median housing cost (logarithm transformed). These variables may reflect the different industrial and socioeconomic conditions across the 29 metropolitan areas in the 2011 AHS. Moreover, in this study, we used the 2010 metropolitan level isolation index calculated by the Spatial Structure in Social Science at Brown University [52]. Isolation index is a measure of segregation that ranges from 0 (i.e., a minority group is not isolated at all) to 100 (a minority group is entirely isolated). Larger values indicate higher segregation. The isolation index for the following racial/ethnic combinations was considered: non-Hispanic whites and non-Hispanic blacks, non-Hispanic whites and Hispanics, non-Hispanic whites and Asians.

### 4.3. Analytic Approaches

Descriptive statistical analysis was conducted to gain a basic understanding of the data and the distributions of household structures by race/ethnicity and suburbia/core city were summarized in order to better depict how household structures distribute across these variables. To examine whether household structure is a determinant of suburban residence, the generalized hierarchical linear modeling was applied to the data.^10^ Since the AHS provides three levels of information, our modeling approach reflects the data structure. We first implemented a null model where no independent variable was considered and then we included individual level and then metropolitan level variables. The potential regional differences are considered with the regional random components. The statistical specification of the full model can be expressed as follows:

$$\eta_{ijr}=\text{log }(\Phi_{ijr}/1-\Phi_{ijr})=\alpha_{000}+\sum \gamma_{0lr}w_{ljr}+\sum \beta_{kjr}x_{kijr}+u_{oj}+\tau_{r}$$

Where η_*ijr*_ indicates the log odds of living in suburbia for the *i*th respondent in the *j*th metropolitan area at the *r*th region, ϕ_*ijr*_ is the odds of living in suburbia for this specific respondent, α_000_ is the intercept, and *u_oj_* indicates the random effect across metropolitan areas. γ_0*lr*_ estimates the association of w_*ljr*_ (covariate *l* in the *j*th metropolitan area at the *r*th region) with suburban residence, and β_*kjr*_ suggests the estimated relationship of x_*kijr*_ (feature *k* of the *i*th respondent in the *j*th metropolitan at the *r*th region) with suburban residence. τ_*r*_ indicates the random effect across regions. Several nested models were obtained and we first included the individual covariates (*x*’s) into the analysis and then added the metropolitan variables (*w*’s) into the regression model. This model helps us to understand if the individual- and metropolitan-level variables explain why a household resides in suburbia.

## 5. Results

Table 1 presents the overall distribution of household structure across residence and nativity status. Several findings are notable. Firstly, married-couple households are the most popular household type, representing 59.9% (53,118). Less than 3% of our samples are multigenerational households (2493). This suggests that married-couple household remains the most popular household type in American metropolitans in contrast to other household structures. One-person households are quite popular, representing approximately 34% (29,874), the second largest household structure. Married-couple households with others comprise 3.5% (3115). This proportion is larger than that of multigenerational households. Most importantly, in the overall proportion of households, suburbia’s most typical household type is the married-couple household.

Secondly, nativity status seems to be associated with multigenerational households. More specifically, among the foreign-born, the proportion of multigenerational households is 5.8%, compared to 2.2% among the native-born. As discussed previously, one plausible explanation is that foreign-born immigrants, usually recent arrivals, tend to live together and depend on their household network to weather economic hardship. By doing so, immigrants gain work through household networks such as family business and also save their living cost. Scholars have postulated that the crowding of recently arrived immigrants appears to be a strategy of life adaptation. For instance, earlier arrivals live with recent arrivals that may live in the living room or kitchen of their cousins’ small house in the city [53,54]. When further taking residence into account, about 5% of the households in central cities were multigenerational households, whereas almost 7% of the households in suburbia were multigenerational households. In contrast, slightly more than 2% of native-born households were found in either central cities or suburbia. Multigenerational households of immigrant groups appear more likely to dwell in central cities than suburbia. This may be explained by the immigrants’ preference for the easy access to urban amenities and ethnic enclaves. The counter explanation would be the presence of barriers for foreign-born multigenerational households to move in suburbia such as segregation in the metropolitan areas. There appears to be no barriers for the native-born multigenerational households to enter suburbia, in contrary to the situation for those foreign-born.

Thirdly, married-couple households prefer to reside in suburbia and this pattern holds for both native-born and foreign-born households. More importantly, more than 70% of the foreign-born households are married-couple household in suburbia, which is almost 10% higher than in central cities. This result seems to support the prior findings that the foreign-born married-couple household population increased in suburbia [3,55]. The discrepancy between central cities and suburbia in the proportion of married-couple households is larger among the native-born. Roughly 63% of the native-born households in suburbia are married-couple and this figure decrease to less than 50% in central cities.

Fourthly, one-person households are more common among the native-born and in central cities. One-person households among the native-born represent 36.2% of the total, compared with 21.3% for the foreign-born. With respect to the residential status of one-person households, they are more likely to appear in central cities than suburbia. This is true regardless of nativity status, but is more conspicuous for the native-born. The percentage of native-born one-person households living in central cities is 45.7%, but the percentage of foreign-born living in central cities is 25.0%. Studies have shown that one-person households prefer central cities over suburbia for their easy access to urban amenities. Finally, married-couple households with others are more prevalent among the foreign-born and in central cities than the native-born and suburbia. In central cities, 5.4% are foreign-born households and 3.6% of the native-born households are married-couple with others.

Table 2 shows the distribution of household structures across residence and race/ethnicity groups. We discuss the main findings as follows. For non-Hispanic whites, more married-couple households live in suburbia (63.2%) than in central cities (47.5%), and slightly more multigenerational households reside in suburbia (1.9%) than central cities (1.3%). On the other hand, one-person households are more dominant in central cities (48.4%) than in suburbia (32.1%), and married-couple households with others appear to be the same in central cities and suburbia.

For non-Hispanic blacks, more married-couple households are observed in suburbia (59.4%) than in central cities (51.3%), but the difference is smaller than that of non-Hispanic whites. More multigenerational households appear in suburbia (3.9%) than in central cities (3.4%). One-person households appear more in central cities (41.4%) than suburbia (33%), and married-couple households with others are more likely to reside in central cities (3.9%) than suburbia (3.7%).

The two non-black minority groups have similar patterns. The proportions of multigenerational households of Hispanics and Asians are higher than those of non-Hispanic whites and blacks, regardless of residence. When comparing the percentages of multigenerational households across central cities and suburbia, more Hispanic and Asian multigenerational households live in central cities than in suburbia but this pattern is reversed among non-Hispanic whites and blacks. This finding seems to suggest that multigenerational households have a different meaning for Asians and Hispanics.

Other patterns across race/ethnicity groups are worth noting. Firstly, married-couple households are dominant group in all races and they tend to more concentrate on suburbia. Proportionately, Asian married-couple households are most likely to reside in suburbia (73.5%), followed by Hispanics (67.6%), whites (63.2%), and blacks (59.4%). Secondly, the similarity occurs for all racial groups in terms of one-person households. One-person households consistently appear more often in central cities than suburbia. Thirdly, the percentage of married-couple households with others was higher in central cities than in suburbia only for minorities and cannot be applied to non-Hispanic whites.

Table 3 presents the results of five logistic multilevel models of suburban residence. Before we discuss the findings, we would like to note that we found substantial variations in suburban residence across metropolitan areas using ANOVA (results not shown but available upon request). The first model used all respondents in the 29 metropolitan areas (pooled model) and it was followed by race-specific models. Given the relatively few Native American and non-Hispanic others respondents, we opted not to report the results of these two race/ethnicity groups but they are available upon request. We summarized the major findings as follows.

First and foremost, household structures were significant determinants in the pooled model. In contrast to the married-couple household (reference group), all other three household structures were less likely to reside in suburbia. Specifically, one-person households were roughly 27% (1 − exp(−0.31) = 0.27) less likely to live in suburbia than married-couple households. For multigenerational households, the odds of living in suburbia decreased by 20% (1 − exp(−0.22) = 0.20) when compared with married-couple households. While the gap between married-couple households and married-couple households with others is the smallest, the odds of moving into suburbia was still 10% lower (1 − exp(−0.11) = 0.11). It should be noted that these relationships were found after all other independent variables were considered.

Secondly, the associations between household structures and suburban residence vary by race/ethnicity. For non-Hispanic whites, only one-person households were less likely to live in suburbia (roughly 30% less) than married-couple households. No significant differences were found for multigenerational households and married-couple households with others. Surprisingly, household structures were not related to suburban residence for non-Hispanic blacks. That is, whether black households live in suburbia is irrelevant to their household structures, and other factors, such as education and age, are more important determinants. With respect to Hispanics and Asians, a married-couple household structure is indicative of suburban residence and the discrepancies between married-couple households and other household structures are larger than those in the pooled model. Take multigenerational households for example. The odds of living in suburbia decreases by almost 40% (1 − exp(−0.47) = 0.38) among Hispanics and by almost 50% (1 − exp(−0.64) = 0.48) for Asians. The largest gap is found between Asian married-couple households and Asian married-couple household with others (1 − exp(−0.69) = 0.50).

Thirdly, nativity status is negatively associated with the odds of suburban residence and this relationship holds for all race/ethnicity groups except non-Hispanic blacks. Generally, the foreign-born were approximately 30% (1 − exp(−0.33) = 0.28) less likely to reside in suburbia in contrast to the native-born. This gap was quite consistent across race/ethnicity groups as the estimates ranged around −0.3. It is worth noting that the number of children in a household is positively associated with odds of moving to suburbia, which echoes the literature suggesting that suburbia attracts married-couple households with children [21]. The relationship between the number of children and suburban residence was the strongest among non-Hispanic whites, followed by Asians. Every additional child increases the odds of moving to suburbia by 20% (exp(0.18) − 1 = 0.20) for non-Hispanic whites and by 15% for Asians. However, similar to the effect of nativity status on suburban residence, the number of children is not associated with residential attainment for non-Hispanic blacks.

Fourthly, other socioeconomic and demographic factors were associated with suburban residence. Home owners were more likely to live in suburbia than renters and this association is universal across race/ethnicity groups. However, there are variations across race/ethnicity groups. For example, educational attainment was not a significant factor for non-Hispanic blacks and household income was not related to residence attainment for non-Hispanic whites. Marital status is only indicative of suburban residence for non-Hispanic blacks. In addition, race/ethnicity is also related to suburban residence in the pooled model. In contrast to non-Hispanic whites, all minority groups, except Native Americans, were less likely to live in suburbia.

Finally, as for the metropolitan level factors, non-Hispanic white households seem to be sensitive to the non-Hispanic white and black isolation. That is, a one unit increase in isolation in a metropolitan area is associated with a 20% decrease (1 − exp(−0.22) = 0.20) in the odds of living in suburbia among non-Hispanic whites. Other isolation indices were not statistically significant and suburban residence of minorities was not dependent on the metropolitan segregation level. The metropolitan level residential stability was associated with household’s suburban residence. That is, higher percentages of owner-occupied housing units and population who did not move in the past year increased the odds of suburban residence. For example, every 1% increase in percent of owner-occupied housing units is associated with a 25% (exp(0.22) − 1 = 0.25) increase in the odds of suburban residence. We would like to note that including other metropolitan covariates, such as unemployment rate and median household income, did not change our findings above.

## 6. Conclusions and Discussion

The findings above were used to examine our hypotheses. Our first major hypothesis is concerned with whether the distribution of household structures differs between suburbia and central cities. Overall, we found difference in the distribution and our three sub-hypotheses were supported. Specifically, we confirmed that one-person households remain common and they are more prevalent in central cities than suburbia (H1a) and that married-couple households are the most popular household structure and they tend to reside in suburbia instead of central cities (H1b). In addition, married-couple households with others are found to be more prevalent in suburbia than central cities (H1c).

Our second major hypothesis focuses on the determinant of residential attainment, particularly the relationship between household structure and suburbia residence and whether this relationship varies by race/ethnicity. After controlling for individual level covariates and metropolitan features, we obtained evidence from the pooled model in Table 3 to support the hypothesis that expects married-couple households to be more likely to live in suburbia than in central cities after controlling for race/ethnicity (H2a). Our findings suggested that, in contrast to married-couple households, all other three household structures were less likely to reside in suburbia. While the race-specific results were not always statistically significant, the patterns followed our expectation. The second hypothesis (H2b) stated that among blacks multigenerational households are more likely to stay in central cities than other household structures. This hypothesis is not bolstered by the analytic findings. More specifically, being a multigenerational household is not a significant determinant of suburban residence, though we found that one-person households are less likely to reside in suburbia than married-couple households. With respect to non-black minorities, we hypothesized that multigenerational households prefer to live in central cities instead of suburbia (H2d). Our results confirm this hypothesis. For Asians and Hispanics, multigenerational households were found to be less likely to reside in suburbia than married-couple households (H2d).

Our findings made contributions to the literature in two ways. Firstly, the multigenerational household structure matters for residential status of non-black minorities, particularly Hispanics and Asians. Hispanic and Asian multigenerational households tend to live in central cities and this relationship holds even after taking other covariates into account. No matter what those households’ economic status is, they are inclined to stay in central cities. One plausible explanation for this finding is that the cultural ideology of “familism” embedded in Hispanics and Asians may play a role in their residential preference [42,56]. Due to collective culture, Hispanics and Asians may be more willing to live in central cities where other extended family members have already settled down. Secondly, the multigenerational household structure does not matter for residential status of whites and blacks. For whites and blacks, the need for the multigenerational households, whether economic or cultural, is not related to the residential choice. Household structure per se is not an important factor when they choose their place to live among whites and blacks.

In addition to the hypothesis testing, our analytical results mostly complied with the finding of the prior studies. Most of the socioeconomic and demographic factors were associated with suburban residence. Home owners were more likely to live in suburbia than renters and this association is universal across race/ethnicity groups. However, other factors such as education, income, nativity status, the number of children, and marital status showed associations varying across race/ethnicity. Educational attainment was mostly significant predictor for suburban residence, but not for blacks. Household income was associated with suburban residence, but not with for whites. Nativity status and the number of children were mostly associated with suburban residence, but similarly, these relationships are not applicable to blacks. Our results of the socioeconomic and demographic factors consist with the prediction of prior studies [21]. However, it may not be universally applicable for all race/ethnicity groups. For blacks, income is more important proxy for socioeconomic status than education.

Our results regarding the metropolitan level factors echo the finding of the white flight thesis. Non-Hispanic white households seem to be only sensitive to the non-Hispanic white and black isolation. Other minorities’ suburban residence was not dependent on the metropolitan segregation level. This finding complies with the white flight arguments [14]. Whites tend to avoid residential areas where there are more people of other minorities, whereas this tendency does not appear for other racial groups.

There are several limitations in this study. First, several important variables were not controlled because of the limitation of the data. Given that the 2011 AHS data lacks data on language proficiency, it is hard to control for the English language proficiency of respondents. As it is certain that speaking English is an important indicator to assimilate into American society and allow living in suburbia, the language variable, English proficiency, should be controlled. Secondly, the analysis is cross-sectional and the causality between household structures and suburbia residence should be further explored with longitudinal data. The question of whether the change in household structure leads to the change in residence also warrants future efforts to answer. This limitation is particularly important when applying the life course perspective [36] to residential attainment research. Thirdly, our findings cannot be generalized to the entire U.S. as the analyses are focused on the metropolitan samples. Fourthly, our findings might, to some extent, underestimate the probability of suburban residence due to the economic recession in 2008 before the data were collected. The economic recession may force some families to move from suburban areas to central cities because of relatively poor resources. However, we would like to note that we did not know the reason for moving in our data, which makes it difficult, if not impossible, to directly evaluate the impact of economic recession. Finally, changing the definitions of central city, suburbia, and household structures may yield different findings.

The findings of this study highlight the importance of household structures in determining residence attainment, a factor that has been overlooked in the literature. Some policy implications can be drawn from this study. In 2016, The Department of Housing and Urban Development (HUD) proposed a new initiative, the Upward Mobility Project, which is designed to reduce poverty, and promote housing stability for families [57]. This project is expected to make families become more self-sufficient, improve children’s outcomes, and revitalize communities. The key finding of this research is that the non-black multigenerational households are more likely to reside in central cities. It suggests that the government should take the family structures into consideration when allocating grant programs. Multigenerational families living in central cities are more likely to be in jeopardy in accessing quality housing. It is not only economic vulnerability of families that we need to consider in designing housing policies and programs, but household structures which may interact with household economy. Careful attention on household structure and race/ethnicity will further the government’s efforts to protect vulnerable families, revitalize neighborhoods, strengthen communities and improve the access to the quality housing.

In addition, as the non-black multigenerational households are more likely to reside in central cities, the housing and neighborhood conditions, overcrowding in particular, should be regularly examined. Given that household crowding leads the detrimental impacts on psychological well-being and marital and household relations [58], housing policy makers need to consider the housing demands of different household types. In contrast, in suburbia, it is important to integrate the married-couple households of minorities into the communities. Under the increased suburban diversity, one of the remaining concerns is the isolation of minority households from affluent whites. On the other hand, number of children is positively associated with the odds of living in suburbia, which may reflect that suburbia is regarded as a better place for children to grow. It becomes important to develop policies that aim to help children in central cities to grow in a safe environment.

## Figures and Tables

**Table 1:** Distribution of Household Structure across Residence and Nativity Status.

		Household Structures				
		
		One-Person Household	Married-Couple Household	Multigenerational Household	Married-Couple Household with Others	Total
**Race**	**Residence**					

Native Born	Central City	10,257	10,876	496	805	22,434
		45.7%	48.5%	2.2%	3.6%	100.0%

	Suburban	16,455	32,153	1140	1603	51,351
		32.0%	62.6%	2.2%	3.1%	100.0%

Total		26,712	43,029	1636	2408	73,785
		36.2%	58.3%	2.2%	3.3%	100.0%

Foreign Born	Central City	1497	3772	403	323	5995
		25.0%	62.9%	6.7%	5.4%	100.0%

	Suburban	1665	6,317	454	384	8820
		18.9%	71.6%	5.1%	4.4%	100.0%

Total		3162	10,089	857	707	14,815
		21.3%	68.1%	5.8%	4.8%	100.0%

Total		29,874	53,118	2493	3,115	88,600
		33.7%	59.9%	2.8%	3.5%	100%

**Table 2:** Residential Status of Household Structure by Race in U.S. Metropolitan Area.

		Household Structure				

		One-Person Household	Married-Couple Household	Multigenerational Household	Married-Couple Household with Others	Total
**Race**	**Residence**					

Non-Hispanic White	Central City	6913	6788	182	395	14,278
		48.4%	47.5%	1.3%	2.8%	100%

	Suburban	14,120	27,797	822	1240	43,979
		32.1%	63.2%	1.9%	2.8%	100%

		21,033	34,585	1004	1635	58,257
		36.1%	59.4%	1.7%	2.8%	100%

Non-Hispanic Black	Central City	2794	3464	230	265	6753
		41.4%	51.3%	3.4%	3.9%	100%

	Suburban	1800	3243	212	201	5456
		33%	59.4%	3.9%	3.7%	100%

		4594	6707	442	466	12,209
		37.6%	54.9%	3.6%	3.8%	100%

Hispanics	Central City	1217	2871	370	390	4848
		25.1%	59.2%	7.6%	8%	100%

	Suburban	1342	4559	404	438	6743
		19.9%	67.6%	6%	6.5%	100%

		2559	7430	774	828	11,591
		22.1%	64.1%	6.7%	7.1%	100%

Asian	Central City	734	1446	109	66	2355
		31.2%	61.4%	4.6%	2.8%	100%

	Suburban	763	2733	138	83	3717
		20.5%	73.5%	3.7%	2.2%	100%

		1497	4179	247	149	6072
		24.7%	68.8%	4.1%	2.5%	100%

Native	Central City	56	53	6	8	123
		45.5%	43.1%	4.9%	6.5%	100%

	Suburban	70	111	16	18	215
		32.6%	51.6%	7.4%	8.4%	100%

		126	164	22	26	338
		37.3%	48.5%	6.5%	7.7%	100%

Others	Central City	40	26	2	4	72
		55.6%	36.1%	2.8%	5.6%	100%

	Suburban	25	27	2	7	61
		41%	44.3%	3.3%	11.5%	100%

		65	53	4	11	133
		48.9%	39.8%	3%	8.3%	100%

**Table 3:** Logistic Multilevel Modeling Results of Suburban Residence by Race/Ethnicity Groups a.

	Pooled Model			White Model			Black Model			Hispanic Model			Asian Model		

	β	SE b		β	SE		β	SE		β	SE		β	SE	
(Intercept)	−87.23	35.70	*	−81.58	31.45	**	−76.43	41.73	†	−65.61	20.44	**	−74.27	28.57	**
*Race/ethnicity*															
Black	−1.09	0.03	***												
Asian	−0.11	0.03	**												
Native	−0.12	0.12													
Others	−1.13	0.21	***												
Hispanics	−0.28	0.03	***												
*Household structure*															
One-person household	−0.31	0.03	***	−0.37	0.04	***	−0.02	0.06		−0.38	0.08	***	−0.24	0.11	*
Multigenerational Household	−0.22	0.05	***	−0.03	0.09		0.24	0.13	†	−0.47	0.09	***	−0.64	0.20	**
Married-couple with others	−0.11	0.04	*	−0.08	0.07		−0.07	0.14		−0.24	0.10	*	−0.69	0.21	**
*Socioeconomic status*															
High school	0.19	0.03	***	0.04	0.05		0.09	0.08		0.08	0.06		0.74	0.16	***
Some college/college	0.08	0.03	**	−0.27	0.04	***	0.14	0.08	†	0.24	0.06	***	0.88	0.14	***
Post-college degree	−0.22	0.03	***	−0.60	0.05	***	0.07	0.11		−0.11	0.13		0.66	0.16	***
Home owner	0.48	0.02	***	0.52	0.02	***	0.42	0.05	***	0.45	0.05	***	0.32	0.07	***
Income (in log)	0.04	0.01	***	−0.01	0.01		0.15	0.02	***	0.07	0.03	**	0.12	0.04	***
*Demographics*															
Household head	−0.02	0.02		−0.04	0.02	†	−0.09	0.05	†	0.04	0.05		0.00	0.07	
Married	0.05	0.02	*	0.03	0.04		0.27	0.07	***	−0.08	0.06		−0.03	0.10	
Foreign-born	−0.33	0.02	***	−0.28	0.04	***	0.08	0.10		−0.39	0.05	***	−0.30	0.08	***
Age	0.01	0.00	***	0.01	0.00	***	−0.01	0.00	***	0.01	0.00	***	0.01	0.00	***
Number of children	0.10	0.01	***	0.18	0.01	***	0.03	0.03		0.06	0.02	**	0.14	0.04	***
*Metropolitan Level*															
W/B isolation c	−0.20	0.07	**	−0.22	0.07	***	−0.08	0.07		0.01	0.05		0.00	0.04	
W/H isolation d	−0.08	0.06		−0.09	0.05	†	−0.01	0.06		0.01	0.05		−0.01	0.04	
W/A isolation e	−0.08	0.09		−0.03	0.08		−0.02	0.09		−0.02	0.07		−0.01	0.07	
% of owner-occupied	0.20	0.09	*	0.21	0.08	*	0.20	0.10	*	0.19	0.07	*	0.20	0.07	**
% of stayer	0.40	0.15	**	0.39	0.13	**	0.33	0.14	*	0.21	0.11	†	0.22	0.10	*
Poverty rate	0.24	0.20		0.30	0.17	†	0.16	0.20		0.23	0.15		0.26	0.15	†
Median housing cost (in log)	5.99	3.90		5.13	3.45		4.87	4.48		4.75	2.33	*	5.62	3.02	†

AIC f	80,682.16			49,825.57			11,090.68			11,538.64			5746.38		
Metro level variance	1.32			1.02			1.06			1.17			0.72		
Region level variance	2.45			2.90			1.24			0.00			0.00		
Pseudo R2 g	0.1813			0.1625			0.2023			0.1510			0.1698		

Notes:

a Due to the limited sample sizes, the regression results for native Americans and non-Hispanic others were not included but they are available upon request;

† *p* < 0.1;

* *p* < 0.05;

** *p* < 0.01;

*** *p* < 0.001;

b SE is Standard Error;

c W/B isolation is the isolation index of non-Hispanic blacks from non-Hispanic whites;

d W/H isolation is the isolation index of Hispanics from non-Hispanic whites;

e W/A isolation is the isolation index of Asians from non-Hispanic whites;

f AIC means Akaike Information Criterion;

g Pseudo R2 is a measure of model diagnostics.

## Footnotes

1 A household is composed of one or more people who occupy a housing unit [22]. Not all households contain families. According to the U.S. Census Bureau, family households consist of two or more individuals who are related by birth, marriage, or adoption, including other unrelated people. Nonfamily households consist of people who live alone or who share their residence with unrelated individuals. In our analysis, we focus on family households, but not on nonfamily households. We decided to use a term, “household” because American Housing Survey (AHS) is a self-reported survey and interviewed one person (not necessarily the head) in a household to collect residence-related information.

2 G.I. stands for Governmental Issue and describes the soldiers of the United States Army. The G. I. Bill indicates the Servicemen’s Readjustment Act of 1944, a law that provided a range of benefits for returning World War II veterans. One benefit of the G.I. Bill was low interest, zero down payment home loans for servicemen. This allowed many American families to move out of urban apartments and into suburban homes [9].

3 Own children are limited to children who have never been married, are under the age of 18 (unless otherwise specified), and are not themselves a family reference person [39].

4 Scholars [23] assert that the recent influx of immigrants from developing countries where extended families are culturally more valued may explain the increasing percentage of extended families among racial minorities and foreign-born immigrants. Drawing on Glick et al. [23], we focus on the race/ethnicity variation of multigenerational households, not the socioeconomic status variation.

5 29 Metropolitan Statistical Areas (MSAs) include Anaheim, CA, Atlanta, GA, Birmingham, AL, Buffalo, NY, Cincinnati, OH, Cleveland, OH, Columbus, OH, Dallas, TX, Denver, CO, FortWorth, TX, Indianapolis, IN, Kansas City, MO, Los Angeles, CA, Memphis, TN, Milwaukee, WI, New Orleans, LA, Virginia Beach, VA, Phoenix, AZ, Pittsburgh, PA, Portland, OR, Providence, RI, Riverside, CA, San Diego, CA, San Francisco, CA, San Jose, CA, St. Louis, MO, Charlotte, NC, Oakland, CA and Sacramento, CA.

6 U.S. Census Bureau defined a family as a household including a householder and one or more people living in the same household who are related to the householder by birth, marriage, or adoption. All people in a household who are related to the householder are regarded as members of his or her family [39].

7 Own children are limited to children who have never been married, are under the age of 18 (unless otherwise specified), and are not themselves a family reference person [39].

8 Other scholars refer to this as an extended family, which is defined as the presence of an individual in a family who is related to the head, but is not a member of the head’s immediate nuclear family [41].

9 The data measured the race/ethnicity of survey respondents. The race/ethnicity of a member of the household does not necessarily represent the entire household.

10 Given the fact that our data are cross-sectional and we did not have one’s residential history, we focus on the distribution of household by residential areas. What we observed in the AHS data is the outcome of choices (and we did not know one’s preferences), which may imply that a household made a decision on their residence. Our analysis cannot make causal inference between household preference (voluntary choice) and residential status.
